# A Study of Dizziness or Vertigo Cases Associated with Inflammatory Bowel Disease (Crohn’s Disease and Ulcerative Colitis) in a Vertigo Outpatient Clinic

**DOI:** 10.3390/jcm14020341

**Published:** 2025-01-08

**Authors:** Teru Kamogashira, Tomoaki Nakada, Kaori Kanaya

**Affiliations:** Department of Otolaryngology, Japan Community Healthcare Organization Tokyo Yamate Medical Center, Hyakunin-cho 3-22-1, Shinjuku-ku 169-0073, Tokyo, Japan

**Keywords:** inflammatory bowel disease, Crohn’s disease, ulcerative colitis, vertigo, dizziness, orthostatic dysregulation

## Abstract

**Background/Objectives**: Dizziness and vertigo are reported in about half of patients with inflammatory bowel disease (IBD), including Crohn’s disease (CD) and ulcerative colitis (UC). Orthostatic dysregulation (OD) is recognized as one of the comorbidities that causes dizziness or vertigo with IBD. Our hospital is affiliated with the Inflammatory Bowel Disease Center, which specializes in diagnosing and treating IBD, so cases with dizziness or vertigo symptoms associated with IBD are sometimes referred to our department, a type of department which is rare in other facilities. The objective of this study is to evaluate IBD cases with dizziness or vertigo symptoms referred to a vertigo outpatient clinic in terms of vestibular function and OD. **Methods**: The subjects were 221 patients who were referred to the vertigo outpatient clinic of our department from March 2021 to September 2024. **Results**: Of the 221 patients, 9 cases had CD and 1 case had UC. OD complications were significantly more common in the IBD group than in the non-IBD group, whereas complications of psychogenic vertigo or migraine were not significantly different between groups, and there was no difference in vestibular dysfunction between groups. OD was a complication in all cases using ustekinumab. **Conclusions**: An orthostatic test will be valuable for diagnosing OD in IBD patients with dizziness or vertigo symptoms.

## 1. Introduction

Orthostatic dysregulation (OD) is a condition in which the body’s autonomic nervous system is unable to properly regulate blood pressure and heart rate when changing positions, such as standing up from a lying position [1]. This results in symptoms such as dizziness, lightheadedness, fainting, palpitations, and sometimes nausea. OD is one of the important diseases in otorhinolaryngology clinical practice for vertigo symptoms [2]. Inflammatory bowel disease (IBD), including Crohn’s disease (CD) and ulcerative colitis (UC), are primarily gastrointestinal disorders characterized by chronic inflammation. IBD is associated with many extraintestinal manifestations that can affect various organ systems, including arthropathies, skin lesions, anemia, osteopenia, osteoporosis, glomerulonephritis, bronchitis, thromboembolisms, pancreatitis, liver diseases, ocular diseases, and neuropathies [3]. Several organ disorders can cause symptoms of dizziness or vertigo, which are observed in nearly half of patients with IBD [4]. OD associated with autonomic nerve dysfunction (neuropathy) is recognized as one of the comorbidities of IBD [4,5]. The prevalence of OD varies across different populations and age groups [6,7]. The prevalences of orthostatic hypotension, orthostatic hypertension, and orthostatic dizziness in a population-based study were 15.9, 1.1, and 4.8%, respectively [8]. OD has also been reported to be associated with autoimmune diseases [9], and a similar mechanism is expected for IBD, which is considered an autoimmune disease. Our hospital is affiliated with the Inflammatory Bowel Disease Center, which specializes in diagnosing and treating IBD; 24,909 outpatients in cumulative total number, 9156 inpatients, and 362 new patients were treated in 2023, which are especially high numbers compared to other hospitals. Therefore, cases with dizziness or vertigo symptoms associated with IBD are sometimes referred to our department, which is rare in other facilities. The objective of this study is to evaluate IBD cases with dizziness or vertigo symptoms referred to a vertigo outpatient clinic in terms of vestibular function and OD.

## 2. Materials and Methods

This was a retrospective study conducted at a single institution. Patients referred to the vertigo outpatient clinic, Department of Otolaryngology at Japan Community Healthcare Organization (JCHO) Tokyo Yamate Medical Center from March 2021 to September 2024 were included in this study. Data on prescription histories, scores of questionnaires regarding psychological symptoms, caloric testing results, and vestibular evoked myogenic potential (VEMP) results were collected. Questionnaires included Dizziness Handicap Inventory (DHI) [10], Hospital Anxiety and Depression Scale (HADS) [11], Self-rating Depression Scale (SDS) (age less than 65) [12] or Geriatric Depression Scale (GDS) (age 65 or over) [13,14], POUNDing (Pulsating, duration of 4–72 h, Unilateral, Nausea, Disabling) [15], Migraine Disability Assessment (MIDAS) [16], and 4-item migraine screener (headache exacerbation in daily performance, nausea, light-sensitivity, and hypersensitivity to odors) [17]. Migraine was diagnosed according to the diagnostic criteria [18].

When OD was suspected based on symptoms, an orthostatic test (Schellong test) [19,20] was performed to diagnose OD. In the Schellong test, blood pressure and pulse were measured immediately after 10 min of supine rest, immediately after rapid standing, and after 10 min of standing. Positive results were defined as a decrease in systolic blood pressure of 21 mmHg or more, a narrowing of pulse pressure of 16 mmHg or more, or an increase in pulse rate of 21 or more [21].

Caloric testing was performed in a darkened room by irrigating the external auditory canal with 20 mL saline solution (4 °C) for 10 s. Caloric nystagmus was recorded using the electronystagmography system (ENG NY-50, RION Co., Ltd., Tokyo, Japan), and CP% was calculated from the maximum slow phase velocity of nystagmus using Jongkees’ formula [22]. An abnormal caloric response was defined by either of the following criteria: (1) left or right canal paresis (CP); CP percentage ≥ 20% [23]; or (2) both side CP; maximum slow phase eye velocity < 10 degree/s [24].

The cervical vestibular evoked myogenic potential (cVEMP) and ocular VEMP (oVEMP) were recorded with the electromyography system (MEB-9404, Nihon Kohden Corporation, Tokyo, Japan) using a tone burst stimulus of 500 Hz at 125 dBpeSPL (rise, 1 ms; plateau, 2 ms; and fall, 1 ms) and a tone burst of 1 kHz at 125 dBpeSPL (rise, 1 ms; plateau, 2 ms; and fall, 1 ms). In cVEMP testing, electromyographic (EMG) activity was recorded from a surface electrode placed on the upper half of each sternocleidomastoid muscle (SCM), with a reference electrode on the side of the upper sternum and a ground electrode on the chin. During the recording, in the supine position, subjects were instructed to raise their heads from the pillow to contract the SCM. The EMG signal from the stimulated side was amplified and bandpass-filtered (20–2000 Hz). The stimulation rate was 5.1 Hz, and the analysis time was 100 ms. Responses to 20 stimuli were averaged three times [25]. In oVEMP testing, subjects lay supine on a bed, with their head supported by a pillow and with surface EMG electrodes placed on the skin 1 cm below (active) and 3 cm below (indifferent) the center of each lower eyelid. The ground electrode was placed on the chin. During testing, the subject looked up approximately 30 degrees above straight ahead and maintained their focus on a small dot approximately 1 m from their eyes. The signals were amplified by a differential amplifier (bandwidth: 0.5–500 Hz). Responses to 20 stimuli were averaged three times [25]. An abnormal VEMP response was defined by the following criteria: asymmetry ratio (AR) percentage ≥ 33% or no response in both ears.

We used a force plate (Gravicorder GW-31, Anima Co., Ltd., Tokyo, Japan; sampling frequency: 20 Hz) with/without a foam rubber layer on the posture platform for evaluating postural sway, recording the sway path of the center of pressure (COP). The foam rubber material was made of natural rubber, with a tensile strength of 2.1 Kgf/cm^2^, an elongation stretch percentage of 110%, a density of 0.06 g/cm^3^, and a thickness of 3.5 cm. Two-legged standing tasks were performed with both arms at the subject’s side under the four conditions: eyes open or eyes closed, with or without the foam rubber. The position of the distal ends of the big toes were 45 degrees apart with the heels of both feet close to each other. The recording time was 60 s or until the subject required assistance to prevent falling. In the eyes-open condition, the subjects were instructed to look at a small red circle 2 m away from where they were standing in a quiet, well-lit room. We measured the mean velocity of movement of the COP for 60 s, which was termed “the velocity”, and the trace by the movement of the COP, which was termed “the total length”.

Excel Microsoft 365 Apps for enterprise (version 2410, Microsoft, Redmond, WA, USA) was used for processing data. Statistical analyses were performed using R version 4.4.2 software (R Core Team; R Foundation for Statistical Computing, Vienna, Austria, 2024) [26] with tableone package version 0.13.2. Data are expressed as mean ± standard deviation. Welch’s *t*-test and Fisher’s exact test were applied to compare the data between the two groups after confirming the Kolmogorov–Smirnov normality test (*p* > 0.05). *p* < 0.05 was considered statistically significant. Multivariate logistic regression analysis was applied to evaluate the association between IBD and OD. Multivariate regression analysis was applied to evaluate the association between postural sway parameters and OD. The statistical power requirements analysis with the group ratios of 0.5 and 0.1, α-error (Type I error rate) of 0.05, 1-β error (Type II error rate) of 0.8, and the sample size ratio of 20 was 10 and 197, which meet the requirements in this study.

This study and the consent procedure were reviewed and approved by the Research Ethics Committee of JCHO Tokyo Yamate Medical Center, approval number J-145. The information for this study was disclosed, and the participants could choose to opt out. An opt-out informed consent protocol was utilized to collect participant data for research purposes.

## 3. Results

Of the 221 patients, 9 cases had CD and 1 case had UC. Case characteristics, results of questionnaires, force plate, and vestibular examinations are shown in Table 1. The age was significantly younger in the IBD group than in the non-IBD group, which reflects the age of onset of IBD. There were significantly more males in the IBD group than in the non-IBD group, reflecting that UC is not gender-specific, but that CD is more common in males. OD complications were significantly more common in the IBD group than in the non-IBD group. Complications of psychogenic vertigo tended to be more common in IBD patients than in non-IBD patients, but the difference was not significant. Migraine complications were not significantly different between IBD and non-IBD groups. Results of questionnaires’ scores tended to be higher for the IBD group than for the non-IBD group, but the differences were not significant. There was no difference in vestibular dysfunction between the IBD group and the non-IBD group. Postural sway evaluated with a force plate showed significant normal values in the IBD group compared to the non-IBD group.

Because the IBD group was significantly more male and younger, a multivariate analysis was performed (Table 2), which showed a significant impact on OD complications in IBD, even after accounting for age and gender.

The multivariate analyses of postural sway parameters regarding OD concerning sex, age, and IBD are shown in Table 3. Sex significantly affected total length (eyes opened) and total length (eyes closed), with more normal values in women. Age significantly affected Romberg rate (velocity, rubber), total length (eyes opened), and total length (eyes closed), with more normal values at younger ages (excluding Romberg rate). The prevalence of IBD significantly affected Romberg rate (velocity, rubber) and total length (eyes closed), with more normal values in IBD cases.

A list of cases with IBD referred to the otolaryngology department is presented in Table 4 and Appendix A. All CD patients had a history of abdominal surgery associated with CD. OD complications were equally prevalent in males and females. Females had more migraine complications than males. This suggests the possibility of gender differences in OD, migraine, and symptom severity. In the Schellong test, pulse rate difference or pulse pressure difference was positive in 2 or 3 cases, respectively, but positive systolic blood pressure reduction was not observed in any cases. There were a few cases that did not meet the criteria threshold but were close to it. No specific medications were prescribed for OD. Prescriptions for OD included antihypotensive drugs (amezinium and midodrine), Kampo (Japanese herbal medicine) (Hangebyakujutsu temmato and Ryokeijutukanto), and diphenidol. There were three cases using ustekinumab, all of which were complicated by OD. Mesalazine, also known as mesalamine or 5-aminosalicylic acid (5-ASA), was prescribed in 8 cases and was not associated with OD complications.

Laboratory test results of patients with IBD are shown in Table 5. Some laboratory test items were not performed in some cases. Nutritional status (total protein, albumin, and vitamin B12), liver function (aspartate transferase (AST), alanine transaminase (ALT), lactate dehydrogenase (LDH), alkaline phosphatase (ALP), gamma-glutamyl transpeptidase (gGTP), total bilirubin (T.Bil), and direct bilirubin (D.Bil)), inflammation (c-reactive protein (CRP)), dehydration (blood urea nitrogen (BUN), creatinine (Cre), sodium (Na), potassium (K), and chloride (Cl)), and anemia (hemoglobin (Hb), red blood cell count (RBC), and hematocrit (Hct)) did not differ significantly between patients with and without OD complications. The zinc testing was performed in all cases with OD complications, but not in some cases without OD complications.

## 4. Discussion

There are multiple possible mechanisms that cause dizziness or vertigo symptoms in Crohn’s disease, which are nutrient malabsorption, side effects of prescription drugs, and chronic inflammation. Severe anemia due to iron deficiency in IBD can also cause dizziness symptoms [27,28,29]. In addition, vitamin B12 or folate deficiency due to malabsorption or poor dietary intake can cause dizziness or fatigue symptoms [30]. Certain medications used to manage IBD, such as steroids or cyclophosphamide, can also cause dizziness as a side effect, likely due to their effects on blood pressure and electrolyte balance [31].

OD is one of the common forms of dysregulation of the autonomic nervous system including the vagus nerve [32,33], and its symptoms are impaired cardiovascular reflexes and orthostatic intolerance, as the body becomes less able to regulate blood pressure and heart rate when changing positions from lying to standing. The chronic systemic inflammatory condition by several cytokines such as tumor necrosis factor-alpha (TNF-α) [34], interleukin-6 (IL-6) [35], interleukin-1-alpha (IL-1α), and interleukin-1-beta (IL-1β) [36,37] is generally observed in IBD, and this condition can damage the autonomic nervous system [38,39] and alter neurological function, affecting blood pressure regulation and heart rate and leading to dizziness or vertigo symptoms due to OD [40]. Past studies have reported autonomic disturbances in IBD, primarily in terms of blood pressure. The systolic blood pressure after tilt was significantly lowered in CD patients compared to controls, both at the first assessment and after seven years [5]. CD patients in remission had preserved cardiac and autonomic function in response to cardiovascular reflex tests and had significantly higher sympathetic-parasympathetic ratio and lower baroreceptor effectiveness and myocardial variables as compared to the healthy controls [41]. UC but not CD patients had greater sympathetic activity than controls in the spectral analysis of heart rate variability to assess cardiac autonomic function [42]. Peripheral sensor-motor neuropathy was relatively frequent in more than one-third of CD patients [4]. Some cytokines that can cross the blood–brain barrier may affect brain regions responsible for balance and proprioception through neuroinflammation by activating microglia [43,44]. Hypothalamic–pituitary–adrenal (HPA) axis may also involve ANS responses [40]. In addition, recent studies suggest an association between IBD and neurodegenerative conditions [45,46], suggesting the importance of the interaction between chronic inflammation and the nervous system. In this study, OD was significantly more common in IBD than in controls, suggesting the importance of OD in dizziness symptoms in IBD.

There are other possible pathways through which chronic inflammation can cause OD. Endothelial cells, which line the blood vessels, are highly sensitive to inflammatory cytokines. Chronic inflammation leads to endothelial dysfunction, reducing the production of nitric oxide (NO), a key vasodilator. NO deficiency can impair blood vessel dilation, affecting blood flow and exacerbating orthostatic intolerance, resulting in reduced vascular responsiveness to positional changes, contributing to the onset of OD in IBD [47,48]. Anemia and hypovolemia are common in IBD due to chronic gastrointestinal bleeding and malabsorption, which can impair oxygen delivery and reduce blood volume. Reduced blood volume contributes to an inability to maintain blood pressure upon standing, a central feature of OD. This combination of anemia and hypovolemia, along with autonomic dysfunction, exacerbates orthostatic intolerance in IBD.

Vestibular dysfunction, potentially linked to autoimmune inner ear disease (AIED), is also a concern in IBD [49,50,51]. The immune dysregulation in IBD can lead to increased susceptibility to autoimmune reactions affecting the inner ear, infiltration of immune cells, and production of antibodies to inner ear antigens that can disrupt the vestibular system, causing dizziness. Biologics and immunosuppressants for managing IBD may also impact vestibular function or cause neurotoxic side effects [52,53]. In this study, the vestibular function was not significantly worse in IBD patients compared to control patients, suggesting that vestibular dysfunction is rare in IBD.

Postural sway, assessed using a force plate, was significantly more normal in the IBD group than in the non-IBD group, indicating the effect of being younger in the IBD group. In the previous study, poor orthostatic tolerance worsens the postural sway parameters as assessed by a force plate [54] and active exaggerated sway improves cardiovascular and cerebrovascular control [55], which are not consistent with the results of this study. IBD complications significantly affected some postural sway parameters, and the IBD group showed significantly better values than the non-IBD group in a multivariate analysis that took sex and age into account. Because the number of cases was small, future prospective studies should validate these findings in larger multicenter cohorts to analyze the impact of multiple factors, including age. Since postural sway evaluates various factors such as psychogenic and central nervous system factors as well as vestibular function, more parameters can be further analyzed, such as the time-frequency spectrum, maximum entropy method [56], and autocorrelation [57].

Recent studies have reported an association between IBD and migraine [58,59,60,61], but further research is needed on a molecular basis. Diagnosis and treatment of migraine in IBD may also be important in terms of vestibular migraine. In this study, the prevalence of migraine did not differ between IBD and controls, which may have been influenced by various factors such as gender and symptoms.

OD was a complication in all cases using ustekinumab, possibly related to the severity of CD. Ustekinumab, which is usually administered by subcutaneous or intravenous injection, is a monoclonal antibody used to treat various autoimmune diseases [62]. In the United States, ustekinumab was approved for psoriasis in 2009 [63], for psoriatic arthritis in 2013, for CD in 2016, and for UC in 2019 [64]. Ustekinumab acts by targeting interleukin-12 (IL-12) and interleukin-23 (IL-23), proteins involved in inflammatory and immune responses. By inhibiting these proteins, ustekinumab reduces inflammation and improves symptoms of these diseases. Recent trials of ustekinumab showed efficacy in treating moderate to severe CD [65]. The UNITI-1 and UNITI-2 trials were randomized, placebo-controlled, phase 3 induction studies evaluating the efficacy of ustekinumab in patients with moderate to severe CD [66]. The UNITI-1 trial included patients who met the criteria for primary or secondary nonresponse to TNF-α antagonists or had unacceptable side effects; the UNITI-2 trial included patients who failed conventional therapy or experienced unacceptable side effects and both trials showed that ustekinumab effectively induced clinical response and remission. UNITI-1 and UNITI-2 patients participated in the IM-UNITI trial to evaluate the long-term efficacy and safety of ustekinumab [67]. Patients who responded to ustekinumab induction therapy maintained clinical response and remission through week 92, supporting the use of ustekinumab as a durable treatment option for severe CD. Based on these trials, the use of ustekinumab is recommended in the treatment algorithm for severe CD, which may reflect the severity of the disease in the data of this study, suggesting an impact of severity on OD complications.

In contrast, mesalazine was prescribed in 8 cases and was not associated with OD complications. Mesalazine is an anti-inflammatory drug used primarily to treat UC and mild-to-moderate CD [68]. Mesalazine inhibits the inflammation of the lining of the gastrointestinal tract by blocking inflammatory mediators such as cytokines and leukotrienes. Mesalazine is usually administered orally or rectally (suppositories or enemas) depending on the site and severity of inflammation [69]. The efficacy of mesalazine in the treatment of mild-to-moderate active CD was equally as effective as a standard dosage of steroids [70]. One systematic review reported that the role of mesalazine in the induction of remission in active CD and prevention of relapse in quiescent CD is still unclear, and more RCTs are needed [71]. Based on these past studies, the presence or absence of mesalazine use is unlikely to reflect the severity of CD. Ustekinumab was prescribed concurrently with mesalazine in two cases, and it was speculated that mesalazine prescriptions were not discontinued even when CD worsened.

In this study, there were no cases of IBD with prominent liver dysfunction and two cases with mildly elevated AST and ALT. There was no significant association between OD and elevated AST or ALT. Hepatobiliary complications are common in IBD, with 30% of patients presenting with abnormal liver function tests and 5% developing chronic liver disease [72]. In a cross-sectional, case-control study, the prevalence of non-alcoholic fatty liver disease (NAFLD) and liver fibrosis did not differ between the IBD and non-IBD groups [73]. These results suggested that the disease is well controlled by treatments, whereas liver function is unlikely to be related to OD.

In the data of this study, there was no significant association between anemia and OD. Impaired stabilization of orthostatic blood pressure and decreased hemoglobin have been reported in chronic kidney disease [74]. Low iron storage is a potentially pathophysiologic factor in both postural orthostatic tachycardia syndrome (POTS) and neurally mediated syncope (NMS) [75,76], suggesting that anemia may affect OD. It is possible that the IBD cases with anemia did not show a significant difference between those with and without OD because they were receiving regular iron supplementation therapy for their anemia, and the values reflected improvement with the therapy.

OD complications were not significantly associated with the zinc testing results based on the available data. However, zinc testing was performed in all cases with OD, but not in those without OD. It is possible that zinc testing tended to be performed more readily in cases of IBD with severe symptoms compared to cases of IBD with mild symptoms. Zinc deficiency in IBD is associated with the development of disease-related complications, and subsequent normalization of zinc levels is associated with improved outcomes [77]. Among the cases in which zinc testing was performed, zinc supplementation therapy was administered in cases of hypozincemia, suggesting that supplementation might be still inadequate. Improvement of various symptoms of IBD with zinc supplementation has been suggested, but whether additional zinc supplementation improves OD should be explored. The prevalence of zinc deficiency was higher in CD patients than in controls, but serum zinc levels were not significantly different between groups [78]. The hypozincemia seen in CD patients is presumably related to accelerated turnover rather than poor zinc absorption [79], suggesting that other underlying treatments, rather than zinc supplementation therapy, may be effective in improving OD.

In the data of this study, no specific medications were prescribed for OD. No particular treatment is particularly effective, and the method considered best for each case was utilized. Treatment strategies for OD include non-pharmacological approaches and pharmacological treatments using antihypotensive drugs [80,81] or Kampo [82]. Non-pharmacological approaches include physical conditioning, hydration with a salt diet, wearing compression garments, and physical counter-maneuvers [83]. Although midodrine has been reported to be effective in previous studies [84,85], a systematic review and meta-analysis of clinical trials have reported a low quality of evidence [81]. Treatment difficulties are speculated, and further new drugs are expected.

Few previous reports have assessed vestibular function in detail in IBD, and this study provides new insights into vestibular function in IBD; however, there are several limitations in this study. Firstly, this was a retrospective study conducted at a single institution. Secondly, the number of cases was too small to analyze differences in symptoms and functions by sex and prescriptions including biologic drugs and immunosuppressants. The risk of bias is high due to the nature of the study, and this study is not complete and should be understood as a preliminary study. Future prospective studies should validate these findings in larger multicenter cohorts. Furthermore, while IBD patients had blood test results for easily deficient items such as Hb, zinc, and vitamin B12, few blood test results were available for the non-IBD group, so comparisons of blood sampling were not possible. Thirdly, nearly half of IBD cases are reported to have dizziness, but most patients do not visit an otolaryngologist, and further research is needed to determine the factors involved in the differences in symptoms. For BPPV, Meniere’s disease, and vestibular neuritis, there may be an autoimmune correlation, but the imbalance has systemic causes, and the help of a neurologist is essential. Additional diagnostic tools such as continuous blood pressure monitoring and neuroimaging could complement the findings. Fourthly, regarding the association of ustekinumab and mesalazine with ODs, each case is assigned to and treated by several physicians, and treatment strategies vary from case to case, thus limiting the ability to make direct comparisons.

## 5. Conclusions

OD complications were significantly more common in the IBD group than in the non-IBD group. In contrast, migraine complications were not significantly different between groups, and there was no difference in vestibular dysfunction between groups. In previous studies, dizziness or vertigo was reported in about half of the patients with IBD, but it was estimated that the patient was unlikely to see an otorhinolaryngologist. IBD patients experiencing orthostatic symptoms may benefit from therapies that target systemic inflammation, address anemia, and optimize hydration status. Autonomic testing will be valuable for the identification and management of OD in IBD, so autonomic testing should be performed regularly in IBD patients with dizziness or dizziness. The importance of multidisciplinary management of IBD patients, especially in collaboration with otolaryngologists and neurologists, is suggested.

## Figures and Tables

**Table 1:** Case characteristics and results of examinations.

	non-IBD (n = 211)	IBD (n = 10)	*p* Value
Sex [Male]	83 (39.3)	8 (80.0)	0.02 *
Age	62.5 ± 18.8	46.7 ± 12.8	0.01 *
OD	23/211 (11%)	5/10 (50%)	<0.01 *
Psychogenic vertigo	98/211 (47%)	7/10 (70%)	0.20
Migraine	36/211 (17%)	2/10 (20%)	0.68
DHI	32.1 ± 21.7	36.4 ± 21.2	0.56
HADS/A	6.9 ± 4.1	9.1 ± 4.9	0.22
HADS/D	6.1 ± 4.0	8.9 ± 3.8	0.06
SDS	42.4 ± 9.8	44.0 ± 5.9	0.48
POUNDing	0.7 ± 1.3	0.8 ± 1.3	0.86
MIDAS	1.4 ± 1.0	1.7–1.2	0.63
Migraine screener [positive]	30/139 (22%)	2/6 (33%)	0.61
Romberg rate (velocity, rubber)	1.8 ± 0.5	1.6 ± 0.2	0.01 *
Rubber ratio (eyes closed, velocity)	1.8 ± 0.8	1.5 ± 0.3	0.04 *
Total length (eyes opened) [cm]	102.1 ± 52.1	76.1 ± 18.4	<0.01 *
Total length (eyes closed) [cm]	145.3 ± 76.6	90.4 ± 26.3	<0.01 *
Caloric testing [abnormal]	106/208 (51%)	2/10 (20%)	0.14
cVEMP 500 Hz [abnormal]	134/205 (65%)	4/10 (40%)	0.17
cVEMP 1 kHz [abnormal]	135/205 (65%)	5/10 (50%)	0.32
oVEMP 500 Hz [abnormal]	167/205 (82%)	6/10 (60%)	0.11
oVEMP 1 kHz [abnormal]	174/205 (85%)	6/10 (60%)	0.06

OD: orthostatic dysregulation; DHI: dizziness handicap inventory; HADS: hospital anxiety and depression scale; SDS: self-rating depression scale; POUNDing: pulsating, duration of 4–72 h, unilateral, nausea, disabling; MIDAS: migraine disability assessment; cVEMP: cervical vestibular evoked myogenic potential; oVEMP: ocular cervical vestibular evoked myogenic potential. *: *p* < 0.05.

**Table 2:** Multivariate analysis of the prevalence of OD concerning sex, age, and IBD.

	Estimate	Std. Error	z Value	Pr (>|z)
(Intercept)	−1.5332	0.70395	−2.178	0.02941 *
Sex	0.2109	0.43231	0.488	0.62565
Age	−0.01073	0.01106	−0.97	0.33224
IBD	1.86558	0.70719	2.638	0.00834 **

*: *p* < 0.05; **: *p* < 0.01.

**Table 3:** Multivariate analyses of postural sway concerning sex, age, and IBD.

**(a) Romberg Rate (Velocity, Rubber)**					**(b) Rubber Ratio (Eyes Closed, Velocity)**				
	**Estimate**	**Std. Error**	**t Value**	**Pr (>|t|)**		**Estimate**	**Std. Error**	**t Value**	**Pr (>|t|)**
(Intercept)	2.1037	0.1385	15.1860	<2 × 10^−16^ ***	(Intercept)	1.7630	0.2166	8.1400	8.05 × 10^−14^ ***
Sex	0.1247	0.0786	1.5850	0.1148	Sex	0.0244	0.1230	0.1980	0.8430
Age	−0.0055	0.0021	−2.6160	0.0097 **	Age	0.0001	0.0033	0.0190	0.9850
IBD	−0.3656	0.1795	−2.0370	0.0432 *	IBD	−0.2882	0.2806	−1.0270	0.3060
**(c) Total Length (Eyes Opened)**					**(d) Total Length (Eyes Closed)**				
	**Estimate**	**Std. Error**	**t Value**	**Pr (>|t|)**		**Estimate**	**Std. Error**	**t Value**	**Pr (>|t|)**
(Intercept)	25.2882	12.2693	2.0610	0.04059 *	(Intercept)	66.4318	18.6957	3.5530	0.000477 ***
Sex	18.8269	6.8645	2.7430	0.00665 **	Sex	35.3345	10.5143	3.3610	0.000935 ***
Age	1.1227	0.1838	6.1070	5.19 × 10^−9^ ***	Age	1.0513	0.2815	3.7350	0.000246 ***
IBD	−14.8325	16.7397	−0.8860	0.3767	IBD	−51.2522	25.4190	−2.0160	0.045134 *

*: *p* < 0.05; **: *p* < 0.01; ***: *p* < 0.001.

**Table 4:** Case series of patients with IBD referred to the otolaryngology department.

**#**	**Sex**	**Age**	**IBD**	**Disease Period**	**Diagnosis**
				**of IBD (years)**	
1	M	49	CD	24	BPPV
2	M	44	CD	17	BPPV after traffic trauma
3	M	49	CD	28	Peripheral vestibular dysfunction
4	M	22	CD	6	Psychogenic vertigo, Delayed endolymphatic hydrops
5	M	51	CD	30	Psychogenic vertigo, OD
6	M	60	CD	27	Psychogenic vertigo, OD
7	M	63	UC	4	Psychogenic vertigo, OD
8	M	58	CD	22	Psychogenic vertigo, OD, Parkinson’s disease
9	F	38	CD	18	Psychogenic vertigo, Migraine
10	F	33	CD	18	Meniere’s disease (probable), Migraine, OD
**#**	**Prescriptions Related to IBD**				**Prescriptions Related to Dizziness**
1	Mesalazine, Budesonide (enema)				−
2	Mesalazine				−
3	Mesalazine, Risankizumab				−
4	Mesalazine, Salazosulfapyridine, Metronidazole, Azathioprine				Amezinium, Domperidone, Hangebyakujutsu temmato, Goshuyu, Saireito
5	Mesalazine				−
6	Mesalazine, Ustekinumab				Betahistine, Ryokeijutukanto
7	Salazosulfapyridine, Mesalazine				Betahistine
8	Salazosulfapyridine, Ustekinumab				Midodrine, Diphenidol, Ryokeijutukanto
9	Mesalazine				Hangebyakujutsu temmato, Goshuyu
10	Ustekinumab				Valproate, Chlorpheniramine, Acetaminophen, Midodrine, Goshuyu, Tokishakuyakusan

#: case number, M: male, F: female, IBD: inflammatory bowel disease, CD: Crohn’s disease, UC: ulcerative colitis, BPPV: benign paroxysmal positional vertigo.

**Table 5:** Laboratory test results of patients with IBD referred to the otolaryngology department.

#	OD	Days	TP	Alb	AST	ALT	LDH	ALP	gGTP	T.Bil	D.Bil	Amy	T-Chol	HDL	LDL	BUN	Cre	CRP	Zn	Fe	VB12	Na	K	Cl	Hb	RBC	Hct
1	−	−33	7.7	4.4	17	19	164	82	17									0.1		78	105				12.3	471	39.8
2	−	68	6.3	3.8	37	66	358	99				65				12	1.1	1		63		140	3.7	108	13.5	449	38.4
3	−	−12	6.8	4	22	26	173	134	115	0.8	0.1	70	122			19	1.02	0.1	86	211		143	4.2	109	14.5	476	44.6
4	−	219	7.2	4.3	21	20	199	53	38	1.2	0	90	98	32	47	6	0.88	0.1				139	4	105	15.8	526	45.1
5	+	−61	7.9	4.1	40	47	135	102	54			101	143	54	56	11	1.03	0.1	75	107	279	139	3.3	98	14.4	479	41.7
6	+	−19	6.9	3.7	22	15	185	90								10	0.8	0.1	75		≤50	142	4.1	104	13.8	464	42.4
7	+	−10	7.6	4.4	24	25	144	77	19			68	240			16	0.98	0.1	113			144	4.2	107	16.4	511	48.3
8	+	−14	7	4.3	26	16	247	53	24	0.5	0	87	181	71	97	25	0.97	0.1	69	112		140	3.9	101	13.6	424	40.9
9	−	−28	7.2	3.5	17	14	130	73	25	0.4		209	193	71	81	9	0.49	0.7	64	56		141	4	105	12	475	39.1
10	+	0	7.9	3.5	20	18	128	76	10			105				7	0.62	0.1	89	42	156	138	3.9	106	11.7	444	37.3

#: case number, Days: days between laboratory test and vestibular test, TP: total protein [g/dL], Alb: albumin [g/dL], AST: aspartate transferase [U/L], ALT: alanine transaminase [U/L], LDH: lactate dehydrogenase [U/L], ALP: alkaline phosphatase [U/L], gGTP: gamma-glutamyl transpeptidase [U/L], T.Bil: total bilirubin [mg/dL], D.Bil: direct bilirubin [mg/dL], Amy: amylase [U/L], T-Chol: total cholesterol [mg/dL], HDL: high-density lipoprotein [mg/dL], LDL: low-density lipoprotein [mg/dL], BUN: blood urea nitrogen [mg/dL], Cre: creatinine [mg/dL], CRP: c-reactive protein [mg/dL], Zn: zinc [µg/dL], Fe: VB12: vitamin B12 [µg/dL], Na: sodium [mEq/L], K: potassium [mEq/L], Cl: chloride [mEq/L], Hb: hemoglobin [g/dL], RBC: red blood cell count [×10^4^/µL], Hct: hematocrit [%].

## Data Availability

The data that support the findings of this study are available from the corresponding author, upon reasonable request with the permission of the research ethics committee.

## Appendix A

**Table A1:** Test results patients with IBD referred to the otolaryngology department.

**#**	**Schellong Test**														**Pulse** **Pressure**			**Systolic** **Diff.**			**Pulse** **Rate Diff.**		**Pulse** **Pressure Diff.**			
	**S**			**D**		**P**	**S0**	**D0**		**P0**	**S10**	**D10**	**P10**													
1	134			89		53	127	97		64	130	95	63		45	30	35	7		4	11	10	15		10	
2																										
3																										
4	109			62		55	108	68		74	105	71	69		47	40	34	1		4	19	14	7		13	
5	94			69		56	107	77		83	102	80	68		25	30	22	−13		−8	27 *	12	−5		3	
6	102			69		80	90	75		94	98	79	98		33	15	19	12		4	14	18	18 *		14	
7	146			91		86	137	98		106	142	98	101		55	39	44	9		4	20	15	16 *		11	
8	134			84		71	123	85		85	126	96	87		50	38	30	11		8	14	16	12		20 *	
9	106			70		62	106	79		81	108	83	72		36	27	25	0		−2	19	10	9		11	
10	97			58		76	85	55		110	86	60	98		39	30	26	12		11	34 *	22 *	9		13	
**#**	**DHI**			**HADS**		**SDS**	**POUNDing**	**Migrane**			**MIDAS**	**Romberg Rate**			**Rubber Ratio**		**Total Length**					**Caloric Testing**				
				**A**	**D**			**Screener**				**(Velocity, Rubber)**			**(Eyes Closed, Velocity)**		**(Eyes Opened) [cm]**			**(Eyes Closed) [cm]**		**L (degree/s)**	**R (degree/s)**		**CP%**	
1	12			4	5	35	0	0			1	1.63			1.45		85.95			97.67		36.5	48		14	
2	56			3	9	39	0	0			1	1.29			1.61		74.03			84.71		13.5	17		11	
3	28			5	7	37						1.53			1.44		66.74			77.39		14	22		22	
4												1.91			1.11		47.11			53.36		20	23		7	
5	16			10	10	48	0	0			1	1.29			1.82		100.03			106.79		11	13		8	
6	64			13	9	48	0	0			1	1.68			1.28		56.01			68.87		26.5	29		5	
7	30			7	4	44						1.8			1.03		98.84			107.68		16.5	21		12	
8	14			10	8	45																29	33		6	
9	64			18	17	53	2	1			2	1.52			1.93		68.85			75.69		8.5	10.5		11	
10	44			12	11	47	3	1			4	1.6			1.8		87.52			141.79		27.5	16.5		25	
**#**	**cVEMP Left 500 Hz**									**cVEMP Left 1 kHz**					**cVEMP Right 500 Hz**						**cVEMP Right 1 kHz**					
	**n13**		**p23**		**Amplitude**			**Area**		**n13**	**p23**	**Amplitude**	**Area**		**n13**	**p23**	**Amplitude**			**Area**	**n13**	**p23**	**Amplitude**		**Area**	
	**(ms)**		**(ms)**		**(µV)**			**(µVms)**		**(ms)**	**(ms)**	**(µV)**	**(µVms)**		**(ms)**	**(ms)**	**(µV)**			**(µVms)**	**(ms)**	**(ms)**	**(µV)**		**(µVms)**	
1	12.2		18		183			2409		14	19.7	185	2605		13	18.4	313			3789			0		2938	
2	10.9		20.8		200			1957		11.8	20.3	91.8	1926		13	18.3	111			1826	12.6	20.2	170		1787	
3	14		21.4		212			3379		12.5	20.9	269	3670				0			2690			0		2591	
4	11.6		20.1		210			2419		12	18	198	2150		12.7	19.5	86.8			1536	12.9	18.3	134		1756	
5	13.2		20		260			3307		12.6	22.5	348	4125		12.1	17.6	192			3257	12.8	18.8	280		2818	
6					0			3917		13.2	18.5	246	3727				0			4403	12.8	16.8	233		4634	
7	14.1		22		429			3937		13.4	20.9	544	4097		13.3	22	446			4397	12.6	20.1	367		4195	
8	17.6		22.8		127			2428		10.4	13.8	110	2361				0			1473					1458	
9	15.3		24.2		236			3026		14.4	22.9	242	3852		14.5	23.1	269			3710	14.7	23.1	187		4186	
10	12.7		20.6		473			4135		13.9	21.1	422	5508				0			5364			0		5278	
**#**	**oVEMP Left 500 Hz**							**oVEMP Left 1 kHz**					**oVEMP Right 500 Hz**					**oVEMP Right 1 kHz**					**VEMP AR**			
	**n1**	**p1**	**base-n1**		**n1-p1**		**Area**	**n1**	**p1**	**base-n1**	**n1-p1**	**Area**	**n1**	**p1**	**base-n1**	**n1-p1**	**Area**	**n1**	**p1**	**base-n1**	**n1-p1**	**Area**	**cVEMP**		**oVEMP**	
	**(ms)**	**(ms)**	**amp. (µV)**		**amp. (µV)**		**(µVms)**	**(ms)**	**(ms)**	**amp. (µV)**	**amp. (µV)**	**(µVms)**	**(ms)**	**(ms)**	**amp. (µV)**	**amp. (µV)**	**(µVms)**	**(ms)**	**(ms)**	**amp. (µV)**	**amp. (µV)**	**(µVms)**	**500 Hz**	**1 kHz**	**500 Hz**	**1 kHz**
1	10.4	12.3	1.21		2.08		8.38	10.7	13.5	0.38	3.11	8.6	10.5	15.6	1.3	4.37	7.97			0	0	7.97	4.3	100.0	37.6	100.0
2	10.2	15.6	7.28		12.8		3.69	9	15.1	6.97	11.2	4.24	10.3	16	2.3	3.53	5.26	9.05	15.6	2.56	4.55	3.27	25.5	33.2	67.5	31.0
3	11.1	14.9	0.93		1.43		4.58	10.9	14.2	1.21	3.85	7.34	10.7	15.5	2.85	4.24	9.82			0	0	5.32	100.0	100.0	16.2	100.0
4	10.2	14	5.7		14.7		15.6	9.05	11.9	12.3	17.7	12.5	10.1	13.5	11.4	14.7	15	9.25	12.3	8.56	16.3	17.5	21.0	9.4	2.1	20.4
5	10.8	15.9	3.11		6.95		6.24	10	15.5	1.7	4.53	9.07	10.8	16.6	2.91	4.3	6.86	10.6	14.4	1	6.43	6.97	14.2	8.2	28.0	29.8
6			0		0		25			0	0	39.9			0	0	17.3			0	0	15.1	NR	13.6	NR	NR
7	10.8	16.8	4.72		10.3		10.8	9.8	16.1	7.63	15.8	10.7	10.9	16.8	4.05	8.73	7.13	9.9	13.5	5.38	9.95	11.1	3.6	20.6	12.4	24.9
8	11.3	13.8	2.23		3.69		7.2	12.5	16.7	4.48	4.26	12.3			0	0	9.25			0	0	8.87	100.0	100.0	100.0	100.0
9			0		0		8.96			0	0	16.7			0	0	4.77			0	0	6.7	3.7	16.8	NR	NR
10	9.9	12.6	1.91		4.08		10.2	9.2	12	0.99	3.08	6.15			0	0	6.74			0	0	5.54	100.0	100.0	100.0	100.0

#: case number, S: systolic pressure, D: diastolic pressure, P: pulse rate, S/D/P(without numbers): blood pressure in the supine position, S0/D0/P0: blood pressure immediately after standing up, S10/D10/P10: blood pressure 10 min after standing up, *: positive for Schellong test, diff.: difference, amp.: amplitude, AR: asymmetry ratio, NR: no response in both ears.
